# Universal primers that amplify RNA from all three flavivirus subgroups

**DOI:** 10.1186/1743-422X-5-16

**Published:** 2008-01-24

**Authors:** Sheryl L Maher-Sturgess, Naomi L Forrester, Paul J Wayper, Ernest A Gould, Roy A Hall, Ross T Barnard, Mark J Gibbs

**Affiliations:** 1 Australian Biosecurity CRC, University of Queensland, St Lucia, QLD 4067, Australia; 2 Centre for Ecology and Hydrology, Mansfield Rd, Oxford Oxfordshire, OX13SR, UK; 3 School of Botany and Zoology, Australian National University, Canberra, ACT 0200, Australia; 4 School of Molecular and Microbial Sciences, University of Queensland, St Lucia, QLD 4067, Australia

## Abstract

**Background:**

Species within the *Flavivirus *genus pose public health problems around the world. Increasing cases of Dengue and Japanese encephalitis virus in Asia, frequent outbreaks of Yellow fever virus in Africa and South America, and the ongoing spread of West Nile virus throughout the Americas, show the geographical burden of flavivirus diseases. Flavivirus infections are often indistinct from and confused with other febrile illnesses. Here we review the specificity of published primers, and describe a new universal primer pair that can detect a wide range of flaviviruses, including viruses from each of the recognised subgroups.

**Results:**

Bioinformatic analysis of 257 published full-length *Flavivirus *genomes revealed conserved regions not previously targeted by primers. Two degenerate primers, Flav100F and Flav200R were designed from these regions and used to generate an 800 base pair cDNA product. The region amplified encoded part of the methyltransferase and most of the RNA-dependent-RNA-polymerase (NS5) coding sequence. One-step RT-PCR testing was successful using standard conditions with RNA from over 60 different flavivirus strains representing about 50 species. The cDNA from each virus isolate was sequenced then used in phylogenetic analyses and database searches to confirm the identity of the template RNA.

**Conclusion:**

Comprehensive testing has revealed the broad specificity of these primers. We briefly discuss the advantages and uses of these universal primers.

## Introduction

Most current molecular assays for flaviviruses use highly specific primers, which may only amplify from one species, or a range of closely related species [1-4]. In a clinical or quarantine setting the presentation and potential exposures, including relevant travel history, are required to generate a differential diagnosis which is required before testing with specific primers. There is a real need to develop broad range PCR assays that can detect all flaviviruses. Kuno [5] reviewed this subject and compared several diagnostic protocols. His recommendation was a two stage process: initially utilizing broad range group-reactive primers to narrow the range of targets, followed by species-specific primers [5].

Many attempts to develop a systematic means for identifying flaviviruses have been made, including serology and non-serology based tests [6-8]. Due to the increased geographic distribution and severity of disease caused by members of the *Flavivirus *genus, this need is becoming more pressing [9].

The first report of a reverse transcriptase-PCR (RT-PCR) for the detection of multiple species was published in 1990, with the use of species-specific probes targeting the nucleocapsid and envelope coding regions from four different Dengue virus genomes [1]. Tanaka [3] published the first universal primer pair specific for mosquito borne flaviviruses in 1993; the YF1 and YF3 primers targeted the NS5/3'UTR of the genome and were based upon the six flavivirus sequences available at the time. Concurrently Fulop [2] designed a degenerate primer pair targeting conserved sites in the NS5 gene. These primers were successfully tested on thirteen different viruses including those in the tick-borne group and flaviviruses with no known vectors. Pierre [4] redesigned the YF 1 and YF3 primer pair previously developed by Tanaka, incorporating redundant bases to expand the range of viruses amplified. The primers EMF1 and VD8 are unable to detect tick borne viruses because they lack the EMF1 motif [4]. In 2005 Gaunt and Gould designed a universal nested PCR, using six primers targeting the E gene, capable of amplifying cDNA from 60 flavivirus strains. The amplification of cDNA was followed by restriction enzyme digestion to identify a range of virus species [7].

The idea of designing primer sets relevant for diseases found in specific geographic regions has also been investigated by several groups. Meiyu [10] developed the DJS and DJA primer set targeting the NS1 gene; these were used in China to detect Dengue virus (DENV), and Japanese encephalitis virus (JEV). Similarly the primers designed by Tanaka (YF1 and YF3 [3] were used to detect flaviviruses in Brazil. However this primer pair failed to amplify Bussuquara virus (BSQV), a virus native to Brazil [11].

Flavivirus detection and taxonomy has recently become more difficult with the determination of the nucleotide sequence of Tamana bat virus (TABV), and Cell fusing agent virus (CFAV) [12-14], and the discovery of Kamiti River virus (KRV). These viruses are currently classified as tentative members of the *Flavivirus *genus [15], even though phylogenetic analysis indicates they are a distant sister group to the other recognised flaviviruses [16]. They pose a problem for detection using PCR since primers depend on sequence conservation. Gaunt and Gould [7] addressed this problem by using a nested PCR and increasing the degeneracy of primers, and demonstrated primers, with more than 200,000 different combinations in solution, were capable of detecting TABV.

In the present study, we identified conserved sites and developed a universal, non-nested primer pair that amplifies cDNA from each of the major subgroups of flaviviruses, and also TABV, under standard reaction conditions. The region of the NS5 gene amplified contained sufficient variability to allow differentiation of individual viruses. We discuss the advantages of this approach, over the known detection regimes for flaviviruses.

## Results

No potentially useful conserved sites were identified in the first complete alignment, utilising all available sequences. However, the sequences of TABV, CFAV and KRV were identified as a divergent cluster, and once removed several conserved sites were found. The Flav100F and Flav200R primers were designed to complement sites in the NS5 gene that begin at residues 8276 and 9062 relative to the YFV genome (NC_002031). The conserved sites encoded amino acid sequences starting at residues 2720 and 2982 in the YFV polyprotein (NP_041726), which do not correspond to any known conserved sites in flavivirus genomes. The primers have relatively low levels of degeneracy, with 8 and 12 different permutations respectively, discounting inosine positions, or with 512 and 48 permutations when inosines are counted as equivalent to four base degeneracy. To compensate for the primer multiplicity, a slightly higher primer concentration (50 pmole per 50 uL reaction) was used in the PCR.

**Figure 1:**
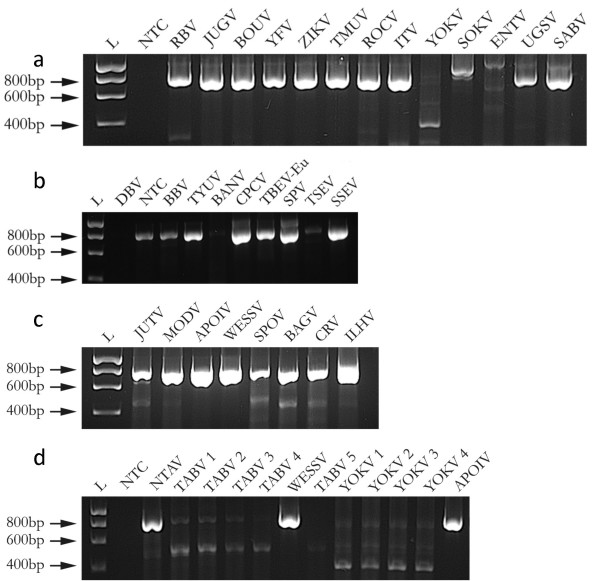
a, b, c) Representative PCR results showing the ~800 bp fragment that was amplified. d) A range of template concentrations was tested for TAMV and YOKV. Reactions marked 1 and 2 have 2 μL template RNA, reactions 3 and 4 have 6 μL RNA; reaction 5 has 10 μL template, reactions 2, 4 and 5 have 4 μL of MgSO_4_. All reactions were performed under identical conditions. (NTC- no template control, L- ladder).

A cDNA product approximately 800 base pairs long was amplified from the RNA of each of the 65 viruses tested (Figure 1). As expected there was variation in product size for some viruses, but products of the correct size were identified for every virus. The sizes estimated after gel electrophoresis corresponded closely with predicted size based on published sequences. When analysed by gel electrophoresis the cDNA products displayed bands of varying intensities at ~800 bp, although for some flaviviruses, products of multiple sizes were visible. Each reaction contained 6 μL of RNA as template, thus the intensity of the product varied, presumably due to template concentration.

All amplified products were sequenced and, on average, sequences from three reactions were used to traverse each cDNA in both directions. Full length sequence was obtained for 55 viruses, and truncated sequence was obtained for DENV2 (771 bp), UGSV (742 bp), BSQV (700 bp), MVEV (684 bp), USUV (675 bp), TYUV (620 bp), TABV (500 bp), YOKV (380 bp). cDNA products of the expected size were obtained from AROAV, BAGV, BOUV and LGTV although reliable sequence data was unavailable; thus these viruses have been excluded from this phylogenetic analysis.

Each product yielded sequence from a flavivirus NS5 gene as shown by BLASTN searches. Flavivirus NS5 sequences occupied the top places in every BLASTN output. The majority of the sequences from the cDNAs differed by 5 to 50 single nucleotide polymorphisms from the closest sequence with the same name in GenBank. Some viruses amplified had no relevant sequence data available on GenBank, the identities of these viruses were further tested by phylogenetic analysis.

**Figure 2:**
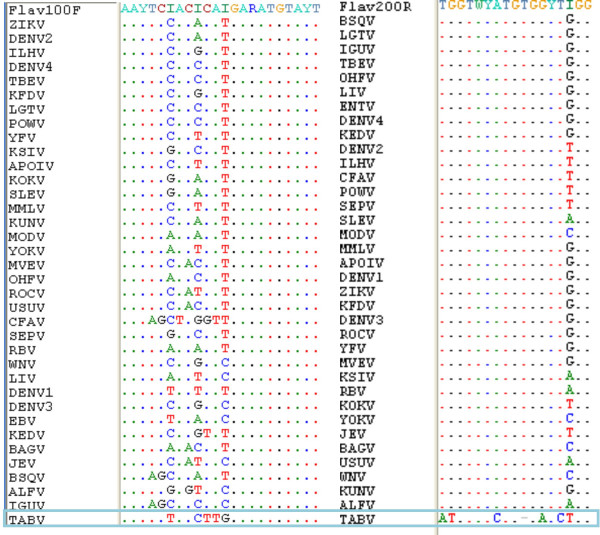
An alignment of the regions targeted by the Flav100F/Flav200R primers. Identities are marked by a dot, gaps are marked by a dash, and nucleotide variants are shown.

The primers were tested on, and amplified cDNA from, 24 of the 27 virus species listed in the mosquito-borne group, 10 of the 12 virus species in the tick-borne group and 13 of 14 viruses in the no known vector group [15]. In total all of the 47 species tested were amplified, seven flavivirus species have not been tested with these primers. cDNA was also amplified from TABV, which was surprising as the available TABV sequences, and those of its closest relatives (CFAV, KRV), were removed from the alignments before the conserved sites were identified. The TABV sequences matched the Flav100F sequence at 18 out of 22 positions and none of the mismatches were located within the last 10 bases of the 3' end of the primer Figure 2. The TABV sequences matched the Flav200R sequence at 10 out of 17 positions and mismatches were located at the 3' end of the primer Figure 2.

Despite this amplification involving mismatching with Tamana bat virus RNA, no cDNA was amplified from the alphaviruses Barmah Forest virus, Ross River virus or the nine respiratory viruses tested: Influenza A virus, Human coronavirus NL, Human coronavirus OC43, Human adenovirus, Human bocavirus, Human rhinovirus 1, 2 or 3 (data not shown).

**Figure 3:**
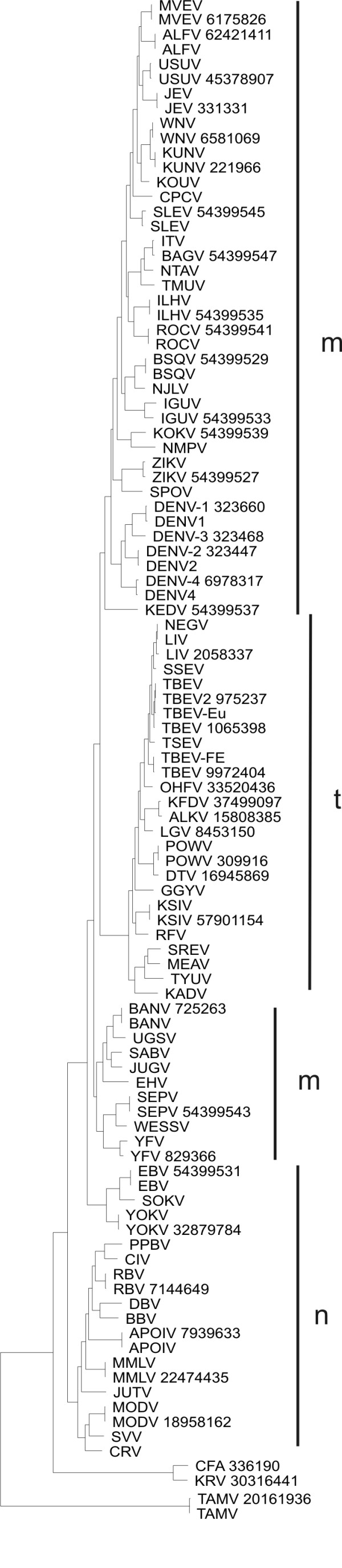
A maximum likelihood tree of the cDNA and references sequence found using an alignment of the 800 base long region. The mosquito-borne (m), tick-borne (t) and no known-vector (n) groups were partitioned as marked.

Phylogenetic trees found using the sequences largely agreed with previously published trees [8,17,18] in that the main subgroups were partitioned and the main known associations between species were found. Sequences from the cDNAs were paired with sequences recognized by the ICTV or reference sequences from GenBank (Figure 3). The LIV cDNA sequence was the only exception, in that it appeared closer to the NEGV cDNA sequence rather than the LIV reference sequence; the LIV reference sequence was the next closest sequence to the LIV and NEGV cDNA sequences. The relationship between LIV and NEGV has previously been determined, thus it is unsurprising these viruses are more closely related to each other than to the reference sequence [19]. All of the reference-cDNA sister groupings in the trees were supported in all bootstrap resamples (100/100); some internal branches closer to the root were also well supported but others were poorly supported. The position of the SLEV sequence appeared to be anomalous, as it clustered with the members of the JEV serogroup rather than ROCV, as previously shown [8]. Phylogenetic analysis of the JEV serogroup shows SLEV to be closely related to members of this serogroup [17,18]. Recent phylogenetic studies using the E-NS1-NS3-NS5 sequence for ROCV and other members of the JEV serogroup shows SLEV to be closer to other members of the JEV serogroup than the ROCV [18,20]. The construction of phylogenetic trees based on shorter sequences, or different regions of the genome leads to different relationships between the viruses in particular the positions of SLEV relative the JEV serogroup and ROCV [8,17,20,21]

## Discussion

We have described a novel primer set capable of amplifying 800 bp from the NS5 genes from almost every recognised member of the genus *Flavivirus*. Since the amplified products represent 8% of the genome, this is sufficient sequence to determine the species of the virus and thus potentially to identify unrecognised flaviviruses. One major problem with degenerate primers is that the concentration of some permutations in the mixture is so small, due to their great multiplicity, that amplification is effectively inhibited. For any given viral RNA target only a proportion of the primer may participate in the initiation of high efficiency extension in the early rounds of PCR. We believe that the redundancy of the Flav100F and Flav200R was insufficient to cause this problem [22].

Traditional serological methods based on neutralisation and fixed cell ELISA have proven effective for identifying flaviviruses and indeed classifying them [23]. However, some were not classified using this technology due to difficulties in interpreting antigenic cross reactivity or failure to identify relatively close antigenic relationships that depend on epitopes encoded by regions of the genome that do not reflect the serological tests. Moreover, serology is time consuming, requires highly experienced personnel and is less precise than nucleotide sequence determination. Using molecular methods, it is now possible to analyse archival material and confirm the identification of tentatively identified flaviviruses. Previous attempts to analyse the entire genus using PCR, have required multiple sets of primers. The capacity of the Flav100R and Flav200R primers potentially to amplify all flaviviruses makes them an invaluable diagnostic and taxonomic tool for virology.

Gaunt and Gould, developed primers targeting the E gene [7]. These primers did not amplify some species including, CIV, CRV, DBV, MMLV, PPBV and TABV [7]. These viruses were all successfully amplified using the Flav100F/Flav200R primers.

Primers targeting the NS3 gene have been developed and tested on a number of viruses including KUNV, JEV and YFV [24]. Bioinformatic analysis using sequence data available at the time, predicted that these primers would be unlikely to amplify products from TBEV thus reducing their usefulness for a genome-wide study [24].

The FU1 and cFD3 primers were tested on a large number of viruses; although six, covering the mosquito-borne KOKV and SOKV, tick borne (KSIV) and no known vector viruses RBV and SVV, were unable to be reproducibly amplified using these primers. These viruses are highly divergent within the three major subgroups currently recognised in this genus [8,15]. The Flav100F/Flav200R primers amplified an 800 bp product from each of these viruses. The NS5 gene has two distinct regions, a methyltransferase and a polymerase [25]. We have targeted regions within two of the more highly conserved functional domains encoded by the flavivirus genome

The primers designed in the present work have been widely tested, but there are six recognised viruses not included in the analysis; the BSL4 viruses, Kyasanur Forest disease virus and Omsk hemorrhagic fever virus, the BSL3 viruses Kedougou virus, San Perlita virus and Yaounde virus and the tentative members of the genus, CFAV and KRV. The primers amplified products from all tested flaviviruses. The ability of these primers to amplify previously 'unidentified' members of the *Flavivirus *genus may demonstrate their capacity to define novel species. The protocol is robust and tolerates a range of template concentrations (greater than five orders of magnitude), primer concentrations, and PCR-cycle conditions (data not shown). The capacity of this reaction to amplify all flaviviruses tested provides a potential tool capable of rapidly identifying endemic and exotic viruses, in a timely, cost effective manner, thus facilitating an appropriate response to epidemic outbreak, or surveys that may result in the discovery of new or novel flaviviruses. These primers also provide researchers with a tool to re-analyse archived samples that may no longer be infectious.

In recent years viruses have been isolated from regions outside their known geographic distribution. JEV was isolated in Australia for the first time in 1995. Until this time the closest location to report human JEV cases was Bali. The 1999 outbreak of WNV in New York reinforces the importance of accurate and rapid diagnosis of exotic viral agents, as the virus was originally mis-diagnosed in serological tests.

Flaviviruses are emerging in new geographic regions as potential epidemic pathogens. Thus, the importance of an accurate, rapid and reliable method for virus identification is becoming increasingly important. A major expansion of arbovirus surveillance and reporting systems has been implemented inNorth America following the appearance of WNV. For example, ArboNet reports surveillance data from humans, mosquitoes, birds, mammals and sentinel chicken flocks and the dataare integrated into a single reporting system [26]. Broad spectrum molecular tests such as that described in thispaper could make a significant contribution to such programmes.

## Conclusion

The changing global epidemiological environment is characterized by incursions of human populations into new environments, increasing overlap of the range of disease vectors with human habitation and concomitant exposure to a wider range of infectious agents [27]. Not only are humans changing land usage patterns and entering new disease environments [28], but rapid transportation of disease agents is constantly increasing between continents. Outbreaks of emerging zoonoses, for example WNV in North America, and the threat of bio-terrorism with novel infectious agents, are no longer remote threats.

The Flav100F and Flav200R primers have the potential to detect emerging, related flaviviruses without prior serological evidence or additional primer design. Our approach should help reduce the confirmation time for viral infections. Rapid detection at the genus level would enable informed policy measures to be implemented and this, in turn, may help disease management.

## Methods

Primers were designed using a strategy similar to that used by Vercruysse *et al *[29]. All available full-length flavivirus sequences were retrieved from NCBI in March 2005. Sequences were sorted using Bioedit [30,31] and aligned using ClustalX [32]. Several divergent sequences were identified by examining neighbour joining trees found using ClustalX and removed from the alignment. Conserved regions were identified by calculating redundancy scores [33], and the average dominant base counts using an early version of the NCSF program and a window length of 20 bases [34]unpublished software, 2005; P. Wayper and M.J. Gibbs]. Average dominant base counts were calculated by summing the number of occurrences of the most common base at each position in the window and averaging those counts across all positions in the window. The distance between conserved regions was taken into account when selecting conserved sites as was the potential for using mixed bases or deoxyinosines, to enhance bonding at variable positions [35]. Standard nucleotides were favoured close to the 3' termini of the oligonucleotides. The primer sequences and their positions relative to the genome of Yellow fever virus (NC_002031) are shown in Table 1. Primers were synthesised by Geneworks (Hindmarsh SA, Australia). The primers target the end of the region encoding the methyltransferase and the start of the region encoding the RNA-dependent RNA-polymerase in the flavivirus NS5 gene.

**Table 1:** Universal primers Flav100F and Flav200R developed and tested in this study.

Primer	Sequence	Binding on YF ref (NC_002031)	
		
		Start	End
Flav100F	AAY TCI ACI CAI GAR ATG TAY	8276	8296
Flav200R	CCI ARC CAC ATR WAC CA	9062	9078

N = A+C+G+T, R = A+G, W = A+T, Y = C+T.

These primers work with Inosines or N included during primer synthesis.

Virus stocks produced in the molecular virology laboratory at the University of Queensland, Australia were prepared from the supernatant medium of infected PS-EK cell cultures (Table 2). PS-EK cells were grown in Dulbecco's Modified Eagle Medium with 10% Foetal Bovine Serum (Gibco, Carlsbad, California), 50U/mL Penicillin and 50 μg/mL Streptomycin (Invitrogen, Carlsbad, California). To remove cellular debris, the supernatant medium was centrifuged at 1,500 rpm for 5 min at 4°C. To increase the concentration, the virus particles were precipitated using a 40% Polyethylene Glycol (PEG) 8000 NTE solution (0.5 M NaCl, 10 mM Tris-HCl, 1 mM EDTA, pH 8.0). The virus-PEG solution was stirred for 16–24 hours at 4°C, centrifuged at 10,000 rpm for 1 hour at 4°C then resuspended in NTE. Viruses tested at Oxford were prepared from the supernatant medium of infected 10% suckling mouse brain suspensions in PBS [36]. Viral RNA was isolated from both sources using RNAqueous kit according to the manufacturer's protocol (Ambion, Austin, Texas).

**Table 2:** List of virus sequences obtained using the Flav100F/Flav200R primer set [42,43].

Virus abbreviation	Virus name	Lab reference	Strain Information	Genbank reference number
ALFV	Alfuy virus	A5-76	MRM392/9	EU303181
APOIV	Apoi	Tc902	kitaoka-> canals ->NIMR	EU303182
BANV	Banzi	Tc802	P12 (11/12/74)	EU303183
BBV	Bukalasa bat	Tc270	BP111	EU303184
BSQV	Bussuquara	Tc836	BE An 4073	EU303185
CIV	Carey Island	Tc960	p10-1215	EU303186
CPCV	Cacipacore	Tc925	Be An 327600	EU303187
CRV	Cowbone Ridge	Tc611	3228	EU303188
DBV	Dakar bat	Tc317	p6 24/4/75	EU303189
DENV1	Dengue virus 1	EF2005		EU303190
DENV2	Dengue virus 2	EF2005	New Guinea C	EU303191
DENV4	Dengue virus 4	EF2005		EU303192
EBV	Entebbe bat	Tc854	221171	EU303193
EHV	Edge Hill	A2-41	c281	EU303194
GGYV	Gadgets Gully	A5-66	CS 122	EU303195
IGUV	Iguape	Tc888	SP An 71686	EU303196
ILHV	Ilheus	Tc837	B52456 (8/9/59)	EU303197
ITV	Israel turkey meningoencephalitis	Tc140	Sent from Israel in 1959	EU303198
JEV	Japanese encephalitis	Tc367		EU303199
JUGV	Jugra	Tc877	P9-314	EU303200
JUTV	Jutiapa	Tc901	JG 128	EU303201
KADV	Kadam	Tc649	ArMp 6640	EU303202
KOUV	Koutango	Tc519	13/11/75 Passage 8	EU303203
KSIV	Karshi	Tc192	30517 (p sm 5)	EU303204
KUNV	Kunjin	A6-60	MRM61C	EU303205
LIV	Louping ill	Tc537	Isle of Mull (pig)	EU303206
SSEV	Spanish Sheep encephalomyelitis	Tc532	87-2617 [42]	EU303207
TSEV	Turkish sheep Encephalitis	Tc530	TTE/80 [42]	EU303208
MEAV	Meaban	Tc647	Brest/Ar/T 715	EU303209
MMLV	Montana myotis leukoencephalitis	Tc770	13302	EU303210
MODV	Modoc	Tc866	3321	EU303211
MVEV	Murray Valley encephalitis	A3-68	1–51 (1992)	EU303212
NEGV	Negishi subtype (LIV)	Tc643	From Dr Shope – P8 11/3/60	EU303213
NJLV	Naranjal	Tc904	25008 22506-8 (Harvard 9/3/83)	EU303214
NMPV	New Mapoon Virus		CY1014 [43]	EU303215
NTAV	Ntaya	Tc554	Original 2/3/72	EU303216
POWV	Powassan	Tc189	Canadian isolate 1968	EU303217
PPBV	Phnom Penh bat	Tc322	Cambodia (A-38D)	EU303218
RBV	Rio Bravo	Tc877	US Bat p9 3360 18/4/68	EU303219
RFV	Royal Farm	Tc959	EG Art 371	EU303220
ROCV	Rocio	TC896	SP H 34675	EU303223
SABV	Saboya	Tc897	IPD/RV 4600 62116	EU303221
SEPV	Sepik			EU303222
SLEV	St Louis Encephalitis	Tc884		EU303242
SOKV	Sokoluk	Tc898	Harvard 12/5/74	EU303224
SPOV	Spondweni	Tc875	30240	EU303225
SREV	Samarez Reef			EU303226
STRV	Stratford virus	B5-11	C338	EU303227
SVV	Sal Vieja	Tc955	78 TWM 106	EU303228
TABV	Tamana bat	Tc885	Trinidad Tr127154	EU303229
TBEV	Tick-borne encephalitis		Neudoerfl	EU303230
TBEV-Eu	Western tick-borne encephalitis	Tc616	Hypr	EU303231
TBEV-FE	Far Eastern Tick borne encephalitis	Tc576	Vasilchenko	EU303232
TMUV	Tembusu	Tc865	N2 revived 14/6/82	EU303233
TYUV	Tyuleniy	Tc310	6017 3 Arch Rock	EU303234
UGSV	Uganda S	Tc767	3/4/8? Isolated sept 1971	EU303235
USUV	Usutu	Tc523	SAR 1776	EU303236
WESSV	Wesselsbron	Tc368	GEN 3 p18 12/9/74 (p17 1972) 17/3/71 rec	EU303237
WNV	West Nile	Tc504	99-349040-31A (NY-99)	EU303238
YFV	Yellow Fever	Tc7	H203410	EU303239
YOKV	Yokose	Tc880	Oita-36	EU303240
ZIKV	Zika	Tc867	MR766 (p4 15/9/76)	EU303241

One-step RT-PCR was performed using Superscript III in a 50 μL volume (Invitrogen, Carlsbad, California) with touch-down cycling conditions [37]. The final primer concentration in the RT-PCR was 1pmol per μL. A 40-min reverse transcription step was performed with incubations for 10 minutes at each of 46°C, 50°C, 55°C and 60°C. Enzyme activation at 94°C for 15 minutes was followed by the touch down PCR. During cycling, denaturation and extension were performed at 94°C for 15 seconds and 68°C for 60 seconds respectively. Annealing occurred for 30 seconds during each cycle, with one cycle at each of the following temperatures of 56°C, 54°C, 52°C, 50°C, 48°C, 46°C, 44°C and 42°C. After the touch down stage, 36 cycles with a 40°C annealing temperature, and then a final extension for 10 minutes at 68°C completed the programme. The reaction was held at 11°C until processing then stored at -20°C.

The specificity of the primers was investigated by attempting amplification from cultures infected with viruses that are not flaviviruses, including Barmah Forest virus, Ross River virus, Influenza A virus, Human coronavirus NL, Human coronavirus OC43, Human adenovirus, Human bocavirus, Human rhinovirus 1, 2 or 3 and RNA from virus free cell cultures.

RT-PCR products were cloned into the pGEM-T easy vector (Promega, Madison, Wisconsin) according to the manufacturer's protocol. Colonies were PCR screened for the presence of an insert. Positive colonies were grown overnight in LB with 1 μg mL^-1 ^ampicillin. The plasmid was purified using a spin column kit (Qiagen, Eppendorf or Invitrogen) according to the manufacturer's protocol. Colony PCRs were performed using a step down protocol as described above although the extension temperature was 72°C (Invitrogen, Carlsbad, California). RT-PCR and PCR products were analysed on a 1% agarose gel containing ethidium bromide, and visualised using a UV transilluminator.

Purified plasmid was sequenced using ABI BigDye Terminator Version 3.1 chemistry, on the AB3730xl sequencing platform. SP6 and T7 promoter primers were used for sequencing. Each virus clone was sequenced twice or more in the forward and reverse directions.

Sequence data were assembled using Contig Express (Invitrogen, Carlsbad, California). Sequences were then compared to the GenBank non-redundant nucleotide database using BLASTN [38]; the programme identified the most closely matching sequences and produced alignments. Species and strain names were matched between the GenBank records and the virus isolates from which template RNA was extracted. RT-PCR reactions were considered to have been successful if the highest scoring alignment was made with a sequence from the expected flavivirus and the correct region of the genome. Publications were traced from the Genbank files to confirm that the sequences had been correctly named. Virus strain names were only used for those isolates where the strain had been identified by the International Committee on Taxonomy of Viruses (ICTV) [15]. If there was no relevant sequence information available in the GenBank database then the identification was based on phylogenetic analysis.

Sequences of known species and strains, identified by the ICTV using their Genbank accession codes, were compiled with the sequences from the amplified products; sequences were then aligned using the default single step progressive method of the program MAFFT version 6.0 [15,39]. Maximum likelihood phylogenetic trees were found for the aligned sequences using the program PhyML [40]; a general time reversible model was used, nucleotide frequencies and the proportion invariant nucleotides were estimated from the data, and variable rates were allowed at different positions with four rate categories. Bootstrap analyses were done using the program PAUP version 4 [41] using the maximum parsimony and neighbour-joining methods.

## Abbreviations

3'UTR: 3' Untranslated region; BSQV: Bussuqara virus; cDNA: complementary Deoxyribonucleic acid; CFAV: Cell fusing agent virus; DENV: Dengue virus; DJA: primer; DJS: primer ; E gene: Envelope protein encoding gene; ELISA: Enzyme Linked Immuno-sorbent assay; EMF1: primer; ICTV: International Committee on Taxonomy of Viruses; JEV: Japanese encephalitis virus; KOKV: Kokobera virus; KRV: Kamiti River virus; KSIV: Karshi virus; KUN: Kunjin virus; L: Ladder; NS1: Non-Structural 1; NTC: No template control; NS5: Non-Structural 5; NTE: a type of buffer; PAUP: a Phylogenetic programme; PBS: Phosphate buffer Saline; PCR: Polymerase Chain Reaction; PEG: Poly-ehtylene glycol; PhyML: Phylogenetic programme; PSEK: Porcine stable Equine kidney cells; RBV: Rio Bravo virus; RNA: Ribonucleic Acid; RT-PCR: Reverse-Transcription Polymerase Chain Reaction; SOKV: Sokoluk virus; SVV: Sal Vieja virus; TABV: Tamana Bat virus; TBEV: Tick-borne encephalitis virus; UV: Ultra Violet; VD8: primer; YF1: Primer; YF3: Primer ; YFV: Yellow fever virus;

## Competing interests

RTB is a Director of Biochip Innovations Pty Ltd.

## Authors' contributions

SLM planned and performed the experiments and drafted the manuscript. MJG designed the primers with assistance from PJW and SLM. MJG, SLM, RH and RB planned the project and edited the manuscript along with NLF and EAG. All authors read and approved the final manuscript.
